# A Randomized Controlled Trial of Short and Standard-Length Consent Forms for a Genetic Cohort Study: Is Longer Better?

**DOI:** 10.2188/jea.JE20110104

**Published:** 2012-07-05

**Authors:** Kenji Matsui, Reidar K. Lie, Tanvir C. Turin, Yoshikuni Kita

**Affiliations:** 1 Department of Preventive Medicine and Epidemiologic Informatics Office for Research Ethics, The National Cerebral and Cardiovascular Center, Suita, Osaka, Japan; 2 Department of Health Science, Shiga University of Medical Science, Otsu, Japan; 3 Department of Philosophy, The University of Bergen, Bergen, Norway; 4 Department of Medicine, The University of Calgary, Calgary, Alberta, Canada

**Keywords:** informed consent, cohort study, ethics, genetics, randomization

## Abstract

**Background:**

Although the amount of detail in informed consent documents has increased over time and the documents have therefore become very long, there is little research on whether longer informed consent documents actually result in (1) better informed research subjects or (2) higher consent rates. We therefore conducted an add-on randomized controlled trial to the Takashima Study, a prospective Japanese population-based genetic cohort study, to test the hypothesis that a shorter informed consent form would satisfy both of the above goals.

**Methods:**

Standard (10 459 words, 11 pages) and short (3602 words, 5 pages) consent forms in Japanese were developed and distributed using cluster-randomization to 293 potential cohort subjects living in 9 medico-social units and 288 subjects in 8 medico-social units, respectively.

**Results:**

Few differences were found between the 2 groups with regard to outcome measures, including participants’ self-perceived understanding, recall of information, concerns, voluntariness, trust, satisfaction, sense of duty, and consent rates.

**Conclusions:**

A short informed consent form was no less valid than a standard form with regard to fulfilling ethical requirements and securing the scientific validity of research.

## INTRODUCTION

Informed consent is an ethical requirement for research on human subjects. In most cases it is also generally agreed that written information on the proposed research should be provided to prospective research subjects, that they must understand this information, and that they should give their valid informed consent. National and international regulations vary with regard to specific requirements on the type of information that should be provided in such written materials. For a number of reasons, the amount of detail provided in informed consent forms (ICFs) has increased over time.^1^^,^^2^ As a result, ICFs have become very long, frequently exceeding 20 pages, though it is often suggested that an ICF should be short enough for subjects to be willing to read it completely.^3^ It is also claimed that excessively long and overly detailed forms can become counterproductive in achieving the original goal, thus increasing the number of “un-informed” subjects.^4^

Several studies have evaluated and attempted to improve the quality of informed consent by modifying the ICF format.^5^ They have mainly focused on ways of scaling and improving the readability of the forms and testing the ability of participants to recall the information provided. However, improving readability and participant memory is not a complete solution for improving understanding and achieving better informed consent.^6^ A few studies have compared the effects of a simplified consent form and a long and detailed standard form. However, most of these studies failed to accurately assess the true impact of ICF length on improved informed consent because they had modified not only the length but also the entire format and readability—and thus the complexity—of statements in the forms.^7^^–^^12^ More importantly, most of these attempts had important limitations in that they were conducted in hypothetical situations and therefore did not assess informed consent in a real research setting. Also, most studies of ICF improvement evaluated patients in the context of clinical intervention trials; thus, their conclusions may not be immediately applicable to a general population that contributes to genetic epidemiologic research.^13^

To address some of these concerns, we investigated the true effects of ICF length on informed consent. Our study was designed as an add-on cluster-randomized controlled trial to an ongoing prospective genetic cohort study. We hypothesized that, as compared with a standard length form, a shorter, simpler ICF would (1) result in better participant recall of key information and superior understanding of the proposed cohort study, and (2) lead to equivalent or better levels of voluntariness, satisfaction, trust in researchers, sense of social obligation, and consent rates to participate in a cohort study.

## METHODS

The Takashima Study is an ongoing prospective genetic cohort study. Recruitment began in 2006 in Kutsuki, Shiga, Japan and was conducted along with the national health-checkup program in that area. Willingness to complete a questionnaire survey on daily nutrition, physical activities, and medical history is the foremost requirement in the inclusion criteria for the cohort study, although participants can specify whether they consent or dissent to other elements of the study, such as donation of blood and urine samples and sample storage for future research.

Two ICFs of different length were developed (Table 1 and Appendix, see SUPPORTING INFORMATION). The standard form is approximately the length of a standard Japanese informed consent document, ie, 10 459 letters/characters (or 335 lines), and comprises 11 pages of *kanji*/*hiragana*/*katakana* of the Japanese language. The standard form was developed and finalized in Japanese and subsequently translated into English by one of the authors (KM) for the purpose of developing a short ICF. The short form comprised 3602 letters/characters (or 152 lines) and 5 pages and was initially developed from the English translation of the standard form by an independent American expert on research ethics and then back-translated into Japanese and finalized by the same author (KM). The finalized standard form and short form shared exactly the same document style, including number of letters per line, illustrations, font style, font size, and same computed levels of word vocabulary and *kanji* characters.^14^ Although the expressions and descriptions in the short form were shortened by eliminating repetition and unnecessary detail and simplified by using plainer expressions (Appendix, see SUPPORTING INFORMATION), both forms contained the following information: research aims, targeted subjects, research methods, targeted disease types, a list of each consent item, anticipated benefits and possible risks, policy regarding feedback on individual research results, policy for data security, voluntariness of participation, short descriptions of the 2 previously specified studies that were scheduled as collaborative projects with which collected samples/data will be shared, the possibility of using samples for unspecified future research, and contact information of the responsible institute and principal investigator. Information on funding support, policy for managing research materials, and detailed information on the 2 collaborating projects was included only in the standard form.

**Table 1.** Comparison of the characteristics of the standard and short informed consent forms

	Standard Form^a^	Short Form^a^	*P* value (χ^2^ test)
Total lines/pages^b^	335/11	152/5	
Total color illustrations	10	10	
Total word count^b^ (letters & characters)	10 459	3602	
Words in introduction and purpose ​ section	611	164	
Words in methods section	1724	742	
Words in collaborative studies section	2841	823	
Words in privacy/confidentiality ​ protection section	1158	450	
Words in voluntariness section	463	241	
Words in benefits section	686	311	
Words in risk/disadvantages section	1098	583	
Words in ethics approval issues	147	0	
Words in research groups description ​ section	819	0	
Words in substudy section	617	148	
Words in contact information section	173	92	
Level of vocabulary^c^ (%)			
>1st grade	14.0	13.2	0.998
1st grade	7.2	8.0	
2nd grade	22.5	24.1	
3rd grade	14.7	14.7	
4th grade	41.5	40.1	
Level of *kanji* characters^c^ (%)			
>1st grade	0.5	0.2	0.991
1st grade	15.2	14.4	
2nd grade	41.5	43.0	
3rd grade	27.9	29.0	
4th grade	14.9	13.3	

^a^Font style (MS PR Gothic) and size (12 points) were the same in both forms.

^b^Numbers of lines, pages, and words were counted using the word count feature of Microsoft Word 2007.

^c^Grade 1 is the level necessary for entering Japanese universities for non-native Japanese speakers who have sufficient knowledge of about a 10 000-word vocabulary and 2000 *kanji* characters, which is almost equal to the level of a native Japanese second- to third-year high school student. Grade 2 is the level of non-native Japanese speakers who have studied Japanese for about 600 hours and completed intermediate Japanese language courses: this is almost equal to the level of native Japanese who have finished elementary education (6 years) with a vocabulary of 6000 words or more and about 1000 *kanji* characters.

A total of 581 individuals were scheduled to attend the health-checkup program. Because our goal was to determine the relative effectiveness of the 2 consent forms at a group rather than an individual level, and because we sought to reduce the possibility of human interaction among individuals living in an intimate neighborhood, the 17 smallest medico-social units (MSUs) within the Kutsuki area were randomly preassigned in a 1:1 ratio to the standard form (the control cohort) or the short form (the intervention cohort). Consequently, the standard form was mailed to the 293 individuals in 9 MSUs, while the short form was mailed to the 288 individuals in the other 8 MSUs (Figure). We then investigated differences between the 2 groups in the following efficacy outcome measures: (1) ICF reading status, namely, the extent to which participants had read the given consent documents in advance, (2) their subjective evaluation of the appropriateness of the length, ease of comprehension, and usefulness of the given form, (3) understanding, including self-perceived understanding and retained knowledge of informed key issues such as benefits and study risks, (4) participants’ concerns, trust, voluntariness, satisfaction, and sense of social obligation, and (5) consent rates.

**Figure.**
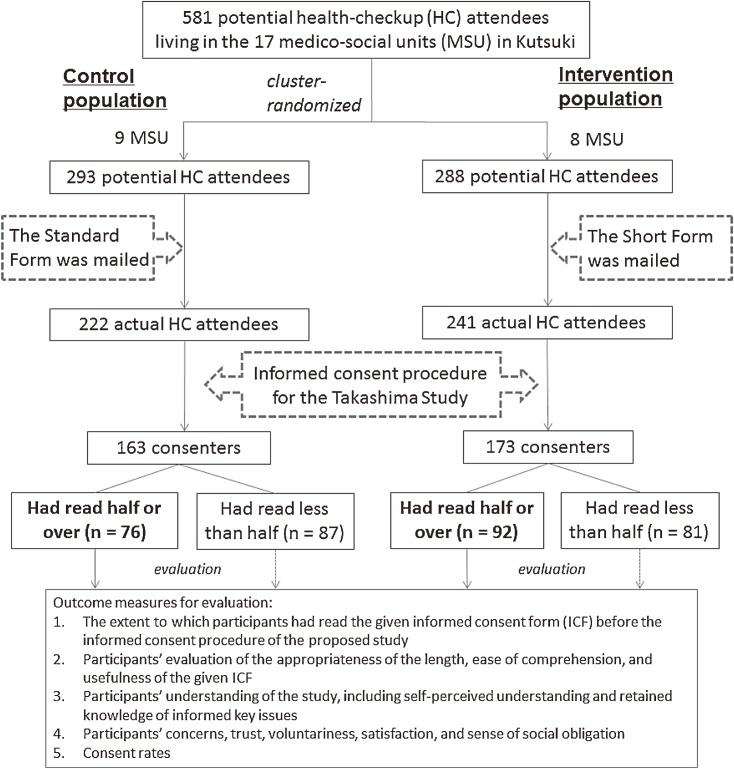
Flows for informed consent and outcome measures for evaluation

The recruitment procedures for the cohort study have been described elsewhere.^15^ Briefly, information documents were mailed in advance to all people scheduled for a health checkup. They were informed that they would be asked to participate in the study at the checkup site and that they should read the information material sent out. At the time of the checkup, the study purpose was again briefly verbally explained to them by a researcher, and people were asked to give their consent to participate in the cohort study. Our study questionnaire assessing informed consent was administered immediately after they had given their written consent to participate in the cohort study. Although the questionnaire was self-administered, the research nurses were allowed to read the questionnaire to an individual when requested to do so.

Data were analyzed using the chi-square test and the Mann-Whitney U-test with the SPSS 14.0J statistical package. Two-sided *P*-values equal to or less than 0.05 were considered to be statistically significant.

## RESULTS

In the control cohort, 222 of 293 (75.8%) individuals attended the checkup, of whom 163 (73.4%) gave basic consent to the Takashima Study. In the intervention cohort, 241 of 288 (83.7%) individuals attended the checkup, among whom 173 (71.8%) consented (Figure). All consenters to the Takashima Study, in both cohorts, assisted with our informed consent study.

The control cohort comprised 60.1% female consenters, and the mean age was 63.7 years. In the intervention cohort, 57.8% were female, and the mean age was 64.5 years (Table 2). More than half of the consenters in both cohorts had less than a high school level education. The baseline characteristics of the 2 cohorts were almost identical.

**Table 2.** Baseline characteristics of participants in the Takashima Study 2006

	Control cohort Standard form (*n* = 163)	Intervention cohort Short form (*n* = 173)	*P* value (χ^2^ test)
	*Number (%)^a^*	*Number (%)^a^*	
Number of women (% female)	98 (60.1)	100 (57.8)	0.666

Age (years)			
<40	13 (8.0)	17 (9.8)	0.633
40–49	18 (11.0)	12 (6.9)	
50–59	25 (15.3)	24 (13.9)	
60–69	36 (22.1)	45 (26.0)	
70≦	71 (43.6)	75 (43.4)	
Mean ± standard deviation	63.7 ± 13.4	64.5 ± 14.3	0.616

Last education (years)			
Junior high school (≦9)	87 (56.9)	89 (53.9)	0.817
High school (≦12)	38 (24.8)	47 (28.5)	
Vocational school (≦13–14)	10 (6.5)	10 (6.1)	
Junior/Technical college (≦14)	4 (2.6)	8 (4.8)	
University (≦16)	9 (5.9)	6 (3.6)	
Graduate School (17≦)	1 (0.7)	2 (1.2)	
Others	4 (2.6)	3 (1.8)	

Occupation			
Company employee	12 (7.7)	7 (4.3)	0.091
Public servant	0 (0.0)	3 (1.8)	
Self-employed	42 (27.1)	61 (37.2)	
Housewife	55 (35.5)	53 (32.3)	
Others	46 (29.7)	40 (24.4)	

Smoking habit			
Current smoker	28 (18.3)	27 (16.3)	0.863
Ex-smoker	24 (15.7)	25 (15.1)	
Nonsmoker	101 (66.0)	114 (68.7)	

Alcohol habit			
Regular drinker	74 (48.1)	91 (54.8)	0.220
Former regular drinker	2 (1.3)	5 (3.0)	
Nondrinker/Occasional drinker	78 (50.6)	70 (42.2)	

Regular prescription drug use ​ (% Yes)	81 (52.6)	102 (60.7)	0.142

How much of the consent form have you read?^b^			
All	34 (21.0)	45 (26.2)	0.503
More than half	15 (9.3)	23 (13.4)	
Half	27 (16.7)	24 (14.0)	
Less than half	30 (18.5)	27 (15.7)	
None	56 (34.6)	53 (30.8)	

^a^Numbers are based on the total number of respondents answering the particular question and may not equal the total number of study subjects.

^b^The *P* value on the Mann-Whitney U test was 0.178.

### ICF reading status

A total of 76 of the 163 consenters (46.6%) in the control cohort and 92 of the 173 consenters (53.2%) in the intervention cohort reported that they had read at least half of the given consent form before the checkup. There was no statistical difference between the cohorts in the reading status of the respective ICFs.

In the analyses below we report only the results obtained from those in both cohorts who responded that they had actually read at least half of the given ICF. We made this decision because the primary purpose of this consent study is to investigate the effects of written consent forms on the quality of informed consent. Thus, it is reasonable to analyze data only from those who could be considered a “reader” of the given forms. Nevertheless, analyses using data from all individuals, ie, including individuals not defined as readers, yielded very similar results.

### ICF evaluations

More than 80% of both cohorts rated the ICF they received as very or moderately helpful in understanding the Takashima Study (Table 3), and most also considered the verbal explanation given before the consent procedure to be helpful. Also, approximately 60% of both cohorts evaluated the length of their respective forms as “appropriate;” however, one-third considered the length to be “more than necessary.” Approximately 80% of both groups also evaluated their respective forms as very or moderately easy to understand.

**Table 3.** Evaluations of written and verbal explanations among participants who reported reading at least half the consent form

	Standard Form (*n* = 76)	Short Form (*n* = 92)	*P* value (χ^2^ test)	*P* value (U test^a^)
	*Number (%)*	*Number (%)*		
How helpful was the consent form in understanding the study?				
Very helpful	9 (11.8)	19 (20.7)	0.179	0.489
Helpful	58 (76.3)	58 (63.0)		
Somewhat helpful	8 (10.5)	14 (15.2)		
Not at all helpful	1 (1.3)	0 (0.0)		
No answer	0 (0.0)	1 (1.1)		

What did you think of the amount of information on the consent form?				
More than necessary	26 (34.2)	35 (38.0)	0.848	0.567
Appropriate	49 (64.5)	55 (59.8)		
Insufficient	1 (1.3)	1 (1.1)		
No answer	0 (0.0)	1 (1.1)		

How easy was the consent form for you to understand?				
Very easy	12 (15.8)	18 (19.6)	0.794	0.996
Easy	51 (67.1)	55 (59.8)		
Difficult	12 (15.8)	17 (18.5)		
Very difficult	1 (1.3)	2 (2.2)		
No answer	0 (0.0)	0 (0.0)		

How helpful was the verbal explanation in understanding the study?				
Very helpful	17 (22.4)	26 (28.3)	0.086	0.100
Helpful	54 (71.1)	62 (67.4)		
Somewhat helpful	4 (5.2)	0 (0.0)		
Not at all helpful	1 (1.3)	0 (0.0)		
No answer	0 (0.0)	4 (4.3)		

^a^“No answer” was excluded from all analyses using the Mann-Whitney U test.

### Self-perceived understanding

Table 4 indicates how both cohorts self-evaluated their understanding of several key issues that were expected to be understood when they participated in the cohort study. The results for most measures of self-perceived understanding were similar between groups, except that the intervention cohort rated their understanding as marginally better than that of the control cohort with regard to the responsible institute and contact information (*P* = 0.063) and the content of contribution (*P* = 0.075).

**Table 4.** Comparison of the comprehension of study participants receiving the standard and short consent forms

		Standard Form (*n* = 76)	Short Form (*n* = 92)	*P* value (χ^2^ test)	*P* value (U test)
***Self-perceived understanding***		*Number (%)^a^*	*Number (%)^a^*		
Do you understand which institution is responsible for the study ​ and whom you can contact with questions?					
Yes		62 (81.6)	84 (91.3)	0.063	
No		14 (18.6)	8 (8.7)		
Don’t know		0 (0.0)	0 (0.0)		
How is your understanding of the purpose of the study?					
Good		40 (52.6)	47 (51.1)	0.991	0.896
Moderate		32 (42.1)	39 (42.4)		
Poor		4 (5.3)	5 (5.4)		
How is your understanding of what you were asked ​ to contribute to the study?					
Good		38 (50.0)	50 (54.3)	0.075	0.310
Moderate		30 (39.5)	40 (43.5)		
Poor		8 (10.5)	2 (2.2)		
How is your understanding of the way to withdraw from the study?					
Good		32 (42.1)	40 (43.5)	0.971	0.815
Moderate		24 (31.6)	28 (30.4)		
Poor		20 (26.3)	23 (25.0)		
How is your understanding of the anticipated benefits of the study?					
Good		32 (42.1)	37 (40.2)	0.752	0.620
Moderate		34 (44.7)	39 (42.4)		
Poor		10 (13.2)	16 (17.4)		
How is your understanding of the possible disadvantages ​ of participation in the study?					
Good		21 (27.6)	25 (27.2)	0.740	0.702
Moderate		29 (38.2)	40 (43.5)		
Poor		26 (34.2)	27 (29.3)		
How is your understanding of the way of managing and preserving ​ the donated samples and data?					
Good		24 (31.6)	26 (28.3)	0.863	0.804
Moderate		31 (40.8)	41 (44.6)		
Poor		21 (27.6)	25 (27.2)		
How is your understanding of the feedback policy regarding ​ the results of future individual analysis?					
Good		24 (31.6)	30 (32.6)	0.781	0.636
Moderate		26 (34.2)	35 (38.0)		
Poor		26 (34.2)	27 (29.3)		
How is your understanding of the explained collaborative studies?					
Good		26 (34.2)	37 (40.2)	0.686	0.576
Moderate		28 (36.8)	29 (31.5)		
Poor		22 (28.9)	26 (28.3)		
How is your understanding of the policy for handling donated ​ samples and data after completion of the study?					
Good		18 (23.7)	31 (33.7)	0.333	0.338
Moderate		26 (34.2)	25 (27.2)		
Poor		32 (42.1)	36 (39.1)		
How is your understanding of the publication policy of the study results?					
Good		22 (28.9)	27 (29.3)	0.633	0.635
Moderate		28 (36.8)	28 (30.4)		
Poor		26 (34.2)	37 (40.2)		
Is your participation in the study voluntary?					
Yes		75 (98.7)	92 (100.0)	0.924	
No		1 (1.3)	0 (0.0)		
Don’t know		0 (0.0)	0 (0.0)		
Do you remember how long the research on participant samples ​ is going to be conducted?					
Yes		35 (46.1)	51 (55.4)	0.226	
No		41 (53.9)	41 (44.6)		
***Retained knowledge***	*True or* *False*	*Number Correct* *(% Correct)^a^*	*Number Correct* *(% Correct)^a^*		
How will the participants’ personal information such as name ​ and address be handled in the study?					
Participants’ name and address will be encrypted.	True	50 (71.4)	61 (74.4)	0.682	
Participants’ name and address can be accessed by anybody.	False	65 (92.9)	73 (89.0)	0.415	
Participants’ name and address will later be open to the public.	False	63 (90.0)	81 (98.8)	0.024	
Participants’ name and address will be immediately deleted ​ so that no individual will become identifiable.	False	56 (80.0)	62 (76.5)	0.608	
What are the benefits of participating in the study?					
Participants may have free treatments in the future.	False	65 (85.5)	81 (93.1)	0.114	
Participants may receive information on the results of individual ​ genetic analysis in the future.	True	43 (56.6)	49 (56.3)	0.974	
Participants may receive monetary rewards in the future.	False	73 (96.1)	85 (97.7)	0.665	
Participants may contribute to future society.	True	46 (60.5)	56 (64.4)	0.613	
What are the possible disadvantages of participating in the study?					
Participants’ personal information might become public knowledge, ​ regardless of the researchers’ intent.	True	31 (44.9)	27 (35.1)	0.224	
Participants may be asked to bear a part of the study expenses.	False	65 (94.2)	75 (97.5)	0.422	
Through genetic analysis research, participants might learn ​ about serious individual genetic disorders.	True	33 (47.8)	40 (51.9)	0.619	
Individual results will not be shared under any circumstances.	False	66 (95.7)	65 (84.4)	0.026	

^a^Numbers are based on the total number of respondents answering the particular question and may not equal the total number of study subjects.

### Retained knowledge (recall)

Similarly, in all the key information tested for retained knowledge, or correct recall, there was no consistent difference between the 2 cohorts (Table 4). As compared with the control cohort, the intervention cohort had a better recall of the issue of personal information protection (*P* = 0.024); however, it had worse recall of the policy regarding feedback on individual results (*P* = 0.026). There was no correlation between the correct recall of any key issue and the corresponding self-evaluated understanding in either cohort.

### Concerns, trust, voluntariness, satisfaction, and sense of duty

Concerning privacy, more than 70% of participants in both cohorts reported little or no concerns about the security of their personal information managed by the cohort researchers, and almost all (>97%) reported that they considered the researchers of the study to be trustworthy.

With regard to voluntariness, while almost all participants in both cohorts understood the voluntary nature of the study (Table 4), about 25% reported that they felt some pressure to participate during the informed consent process. However, more than 90% of participants in both cohorts had high satisfaction with the whole explanatory process.

Importantly, almost all participants in both cohorts thought that the proposed study seemed more or less useful to themselves (>93%) and to society (>97%). Also, there was a marginal trend (*P* = 0.075) for the control cohort, as compared with the intervention cohort, to consider participation in the study as a charitable activity rather than as a social obligation.

### Consent rates

There was no statistical difference between the 2 cohorts in consent rates for the following 12 consent items: participation in the medical interview and questionnaire studies; allowing National Health-Checkup (NHC) data to be used for the Takashima Study; participation in providing a urine sample collected primarily for the NHC and donating a new blood sample to the study; allowing blood and urine sample preservation for 20 years; participation in donating a DNA sample for research analysis; allowing DNA sample preservation for 20 years; participation in a re-investigation every 5 years; allowing preservation and utilization of unlinked anonymized samples/data for another 20 years after completion of the Takashima Study; authorizing the principal investigators to examine and use medical records and other medical documents in the 20-year follow-up survey; allowing participant data to be provided to the *Tougoukenkyu* (The Existing Cohort Combine), of the Japan Arteriosclerosis Longitudinal Study (JALS-ECC); allowing samples/data to be given to the Japan Multi-Institutional Collaborative Cohort Study; and allowing samples/data to be given to various unspecified future (collaborative) research projects.

## DISCUSSION

This cluster-randomized controlled study of a standard-length versus a short informed consent form was conducted in the real research setting of a genetic epidemiologic cohort study and showed relatively high-quality “informed consent” among those receiving the short form. More importantly, the short form compared favorably with the standard form in almost all outcome measures, including participant reading status, evaluation of the ICF, self-perceived understanding, levels of participant recall, concerns, trust, voluntariness, satisfaction, sense of duty, and consent rates.

Berger et al analyzed 87 ICFs used in actual clinical trials for cancer patients in Norway from 1987 to 2007 and found a significant increase during that time period in the number of text components, especially text regarding formalities, eg, legal and financial matters.^16^ A very likely reason for such a marked increase in the length of forms is legal indemnification of research institutions that seek to avoid the potential liabilities of research.^4^^,^^17^ However, such language has little to do with the interests of research subjects. In addition, too much such attention on legal accountability can increase the length of an ICF and add to preparation costs, in terms of the people, time, and money concerned, for both researchers and research subjects. Such attention does not contribute to achieving the true goal of informed consent, namely, fully voluntary, well-informed, and satisfactory participation based on an improved understanding of a proposed study.

While our hypothesis that the short form is superior to the standard form was not fully confirmed by the present findings, our results suggest that short consent forms are at least equivalent in function and value to longer, more detailed forms. White et al indicated that research subjects sometimes prefer detailed information to abbreviated information.^18^ However, although a longer form might be preferred by some research subjects, if asked to choose among 2 or 3 forms of different length, a longer form is not certain to be ethically preferable to a shorter one. Dresden and Levitt reported that information retention was better among individuals who received a shorter form than among those who received a more detailed form.^7^ Similarly, other studies have reported that understanding of a detailed explanation was worse than that after a simplified explanation.^8^^,^^17^ These previous findings are in conformity with our results. Taken together, these findings indicate that providing less information in a shorter, simplified form is ethically acceptable, and preferable in practical terms, as compared with including more information in a standard form. Although we acknowledge that our results might be partly attributable to the relatively small sample size of our study, the absence of any clear trend regarding a difference between the 2 cohorts is the most important finding. Currently, most research ethics committees are conservative with regard to informed consent, that is, they assume that more detailed, more complicated information is always ethically better for research subjects. However, empirical data indicate that this assumption may not be true and that shorter forms may be ethically equivalent or sometimes superior to longer, standard forms.

The nature of true understanding remains unknown, despite the numerous studies of comprehension that have attempted to measure it.^15^^,^^19^^–^^21^ Because there is no gold standard that can be used to measure true understanding per se, evaluating the understanding of research subjects is a methodological challenge for all such studies. Fundamentally, self-perceived understanding may not be equivalent to true understanding. Yet, at the same time, understanding measured as the extent to which individuals can correctly recall information might also differ from true understanding.^22^^,^^23^ Therefore, to some extent, both measures of understanding will remain necessary in comprehension studies.

Our study also revealed a dilemma in the attempt to identify better ways of improving the informed consent process. We found that only half of both cohorts had actually read at least half of the documents before the informed consent procedure in the study. Although recommendations for learning materials suggest that people are more likely to read shorter rather than longer documents,^3^^,^^24^ our study found no difference in participant reading status between the 2 cohorts. Thus, the apparent length of an ICF seems to have little effect on motivating people to read it. If a large proportion of potential participants do not even try to read a given ICF, any attempt to improve the quality of the text in the form would not actually substantially improve understanding. This also raises ethical concerns for research, especially when research subjects are exposed to much higher risks than in a genetic cohort study,^25^ if they have not read a given ICF but participate without being aware of those risks.

Our findings also highlighted an important ethical controversy over voluntariness, which is 1 of 4 essential attributes of valid informed consent. Although our participants clearly knew that participation in the proposed genetic cohort study was entirely voluntary, many also felt some pressure to participate. As explained in the Nuremberg Code, voluntary decision-making must involve an “exercise of free power of choice, without the intervention of any element of force, fraud, deceit, duress, over-arching, or other ulterior form of constraint or coercion.”^26^ However, voluntary choice does not mean “that it cannot be influenced by a variety of factors, including preexisting characteristics of the individual deciding, aspects of his or her situation, and the desires and actions of third parties.”^27^ Also, not all types of pressure are an undue influence or negate the voluntariness of consent. Indeed, 99% of our participants indicated high trust in the researchers, over 90% had high satisfaction with the overall informed consent process, and half felt a sense of social duty to participate. Such increased trust, satisfaction, and sense of duty to others are inevitable and necessary pressures to achieve ethically and practically successful research recruitment.^28^ Ironically, if these pressures also create an undue influence that diminishes the voluntariness of consent, any attempt to improve informed consent as a whole cannot avoid reducing voluntariness. Because the nature of the voluntariness requirement has not been fully explored, further research is needed to clarify the relationships between voluntariness and pressure to participate.

This study has several limitations. First, as the parent study was a genetic epidemiologic study that involves much less, if any, physical risk than typical clinical trials, the findings may not be immediately applicable to other studies, including clinical trials. Second, the 5- to 8-minute verbal explanation given right before the informed consent process at the health-checkup site might have affected the understanding of our study participants, though the amount and complexity of information given verbally were very limited—much less than that included on the shorter consent form. Third, information on funding support was not provided on the short form, which might raise ethical concerns that the absence of such information might diminish participant understanding of the possible risks of a proposed study. However, several consent studies found that when financial considerations were the primary motivation for participating in clinical trials, individuals’ understanding of the risks was not adversely affected.^29^^–^^31^ Nevertheless, there is a need for further research to determine if and how disclosing (or not disclosing) a financial relationship in a proposed observational study, such as a genetic cohort study, might affect participant understanding and the subsequent quality of informed consent. Fourth, because this study was conducted in a rural area where people are generally considered more cooperative, and because the research team and municipal authorities had long had a good relationship, the results might differ if the study were conducted in an urban area or in an area without such longstanding good relationships. Presumably, if we assume that the main reasons for not participating in a genetic cohort study are lack of time and the time-consuming nature of the study,^32^ a short form might be more effective in practice and ethically preferable in such areas.

This study also has important strengths, the most significant of which is that we conducted this consent study in an actual research situation. In general, obtaining approval for add-on studies of the informed consent process has been difficult because many institutional ethics committees do not want researchers at their institutions to depart from the standard approach. However, our ethics committee accepted both our previous empirical data and our hypothesis and offered us a chance to prove the hypothesis. Another strength of the study is that we separated the development process of the short form from that of the standard form, thereby reducing possible bias in the development of the short form.

In conclusion, this study showed that ICF length does not materially affect the quality of informed consent or consent rate, which suggests that researchers who seek to maximize these 2 goals simultaneously should use a short form that is no less valid than a detailed, standard form and sufficiently satisfies both ethical requirements and practical needs. Also, the unreflective belief that more information is always better than less information should be carefully reviewed before it is used as the ethical basis of research practice and ethics reviews.

## SUPPORTING INFORMATION

Appendix is available on the journal's website at http://dx.doi.org/10.2188/jea.JE20110104.

eAppendix.
