# Supplementary material for: A Randomized Controlled Trial of Short and Standard-Length Consent Forms for a Genetic Cohort Study: Is Longer Better?
Source: J Epidemiol. 2012 Jul 5;22(4):308–16. doi: 10.2188/jea.JE20110104 (PMC3798649; doi:10.2188/jea.JE20110104)
Supplement: eAppendix. [file je-22-308-s001.pdf]

Appendix. Sample of differences in wording (in Japanese) between standard and short informed consent forms (excerpted from the Benefits and Risks section)

Standard Form

研究協力者に発生し得る利益および不利益について

（1）利益

この研究がもたらす成果は、将来、さまざまな医療政策や保健指導・対策などの形で社会全体の健康増進のために役立てられます。研究成果はご提供いただいた方々の情報を集団として統計処理し、学会、学術誌面、あるいはインターネット上において個人が分からない形で公表を行い、社会全体に還元していく予定です。

　健診受診者には、高島市と協議の上決定した追加検査項目（動脈硬化度検査 ABI/PWV、尿中ﾅﾄﾘｳﾑ・ｶﾘｳﾑ・ｸﾚｱﾁﾆﾝ、HbA1c）の結果をお返ししますが、研究参加者には、腹囲、インスリン値、高感度 CRP 値、BNP 値の結果も追加してお返しします。また今後も原則的に、この研究のなかで、診療・治療を行う上で有益・重要であると判断される結果が万一見つかった場合は、説明と助言と一緒にその結果をすべてご本人にお返ししていきます。遺伝子分析の結果についても同様です。

　ただし、この研究では、現時点ではどのような意味・作用があるのか、また、どのような病気と関連しているのか、ということが全く分かっていない項目および遺伝子（DNA）について分析を行っていくため、研究による分析結果が診療上重要かどうか判明するまでには何年もの歳月がかかると予想されます。また、ご本人がすでに亡くなられている場合は、これらの結果は返却いたしませんので、ご了承ください。

　一方、この研究にご協力いただいた場合に、研究の結果として、将来特許権などによる何らかの金銭的利益が生じることがあった場合でも、あなた個人に還元される金銭的利益は一切ありません（こうした権利は国や研究機関などにゆだねることになります）。

⑵不利益

　この研究への参加にあたり、皆さまへの金銭的負担はありません。

（ア）身体的不利益

　ご協力いただける方からは、健診時の採血とは別に、16～18mlの血液を採取させいただきます。採血に伴い、不快（痛み・内出血など）、稀な合併症（細菌感染、神経損傷など）または事故（針折れなど）が生じることがありますが、健診時採血の延長で採血するため、針刺し回数が健診に比べて増えたり、こうした合併症や事故が起こる可能性は、健診の採血にともなってこれらが生じる確率を上回ることはありません。

（イ）社会的不利益、情報に関する不利益

　ご提供いただいた「あなた」に関する資料・試料および情報の管理は、「個人情報保護の方法について（5㌻下欄）」に記載したとおり、厳重に行います。しかし、このように管理していた場合であっても、悪意ある第三者によって管理情報が盗まれたり、漏れ出たりといった事件がまったく起こらないと言い切ることはできません。とりわけ遺伝子（DNA）の分析を行うことにともない、「あなた」に返却した遺伝子（DNA）分析結果を盗み見た悪意ある第三者が、例えば、結婚・雇用・生命保険契約上での差別や何らかの不利益を生じさせる、などの可能性はゼロではありません。万一こうした情報の盗難や漏えいが生じた場合は、社会的な不利益（例えば就職・生命保険加入上の差別）をこうむる可能性があります。しかし、高島研究の研究者に明らかな責任が無い場合の情報盗難・漏洩については、滋賀医科大学としては責任を負いかねますので、どうかこのことをご理解の上、研究へのご協力をお願いします。

　また、「あなた」の血液を分析したり遺伝子多型を明らかにするということは、あなたやご家族の病気について明らかにしてしまうことを意味します。例えば、血液検査から「あなたは重大なウイルス疾患にかかっている」とか、「あなたは大腸がんになりやすい遺伝子をもっている」ということが判明することがあります。また、「あなた」が大腸がんになりやすい遺伝子をもっている場合、あなたの「血縁者」も大腸がんを発症しやすい遺伝子をもっている可能性が高くなります。研究における分析結果の返却を希望される場合は、このように自分と血縁者の病気や遺伝的体質を「知る」ことに伴う精神的ストレスが、皆さま自身とご血縁者に生じることがあります。そのため、知ることに伴う利益・不利益についてあなたご自身が十分にお考えになった上で、こうした分析結果の返却をご希望になるかどうかを、最初のご協力意思確認時にご表明ください。返却を希望されない方には、将来にわたり、この研究で行う分析の結果について、改めてご要望が無い限り当方から返却することはありません。

Short Form

研究参加者の利益

■ 研究成果は、学術専門誌等に個人が分からない形で公表し、医療政策の発展など、社会のために役立てます。

■ 研究参加者個人への金銭的な利益は一切ありません。

■ 健診受診者全員に、高島市と協議の上決定した追加検査項目の結果をお返しすると共に、研究参加者には、腹囲、インスリン値、高感度 CRP、BNP の結果をお返しします。

■ この研究のなかで、診療や治療にとって重要であると判断される結果が万一見つかった場合は、説明・助言とともにその結果を参加者ご本人にお返しします。遺伝子分析の結果も同様です。ただし、分析結果が診療上重要かどうか分かるまで何年もかかります。また、ご本人がすでに亡くなられている場合、結果は返しません。

研究参加にともなう不利益・危害の可能性

■ 研究参加者への金銭的負担はありません。

■ 参加者の個人情報は外部に漏れないよう管理します。しかし、厳重に保護された個人情報に悪意ある第三者が侵入し、外部に漏らす危険を完全に防ぐことは無理なため、情報の盗難・漏えいの危険性はゼロではありません。

■ 盗難・漏えいが起こった場合、社会的な不利益(例えば、就職や生命保険加入での差別)を受けるかもしれません。しかし、この研究の研究者に明らかな落ち度が無い場合の個人情報の盗難・漏えいについては、個々の研究者および滋賀医科大学としては責任を負いかねますので、その点についてご了解の上、研究へのご協力をお願いします。

■ また研究の性質上、将来例えば、血液検査から「あなたは重大なウイルス性疾患にかかっている」とか、「あなたは大腸がんになりやすい遺伝子をもっている」ということが分かることがあります。「あなた」が大腸がんになりやすい遺伝子をもっていた場合、あなたの「血縁者」も大腸がんを発症しやすい遺伝子をもっている可能性が高くなります。

■ 従って、分析結果の返却を希望された場合、自分と血縁者の病気や遺伝的体質を「知る」ことに伴う精神的ストレスが生じる可能性があります。

Appendix. Samples of differences in wording between standard and short informed consent forms (excerpted from the Benefits and Risks section)

STANDARD FORM

Potential Benefits and Disadvantages to Study Participants

(1) Benefits

The results of this study could benefit society in the future through various medical policies, health counseling and health initiatives. We will statistically analyze the information after it is made anonymous and combined and later publicly announce the results to society through academic conferences, academic journals and the Internet, using a format that retains participant privacy.

We will provide to all health-checkup attendees the results of the supplementary test items decided in consultation with Takashima City (ABI/PWV test for arteriosclerosis; urinary sodium, potassium and creatinine levels; and HbA1c levels). Participants in this study will also receive results on waist circumference, insulin levels, high-sensitivity CRP levels and BNP levels.

If any results from this study are judged in the future to be significant or beneficial for the medical care or treatment of an individual participant, we will provide all the information to him/her with an explanation and counseling. This also applies to genetic (DNA) testing results.

This study will analyze genes (DNA) and other biological materials. At this point in time, it is unknown what diseases or disorders they might be related to, and what meaning or consequences they will have. It can be expected to take years to determine whether or not the results from this study will be significant for individual medical care. Also, please note that we will not release the results of a participant who has died.

If, as a result of this study, any financial benefits should be generated in the future through patents or other means, you will not receive any proceeds because the rights for those financial benefits belong to the national government and/or research institutions.

(2) Disadvantages

There will be no financial burden or cost to you for participating in this study.

(A) Physical disadvantages and risks

We ask that study participants allow us to draw 16-18 mL of blood for our research purposes in addition to the blood taken during the physical examination for the health-checkup program. The process of drawing blood may cause discomfort (such as pain or internal bleeding), rare complications (such as bacterial infections or nerve damage) and accidents (such as bent needles), but the likelihood of these events or an increase in the number of needle insertions is no greater than when your blood is taken in the health-checkup program, since this process is conducted at one time.

(B) Social and informational disadvantages and risks

We strictly control information, materials and samples collected from you, the participant, as explained in "On Methods of Personal Information Protection" (the lower half of page 5). However, we cannot absolutely guarantee that no incidents will occur in which a malicious third party steals or leaks this controlled information. With genetic (DNA) test results in particular, there is the possibility of a malicious third party illicitly viewing the results to use against you (eg, in marriage, employment or health insurance contract negotiations). If

this type of information theft or leak occurs, there is a possibility you will incur social disadvantages (eg, discrimination in job hunting or life insurance enrollment). The Shiga University of Medical Science is not responsible for personal information theft or leaks if the Takashima Study researchers are not clearly responsible. We ask for your understanding of this matter when participating in our study.

When analyzing your blood and identifying genetic (DNA) polymorphisms, we could identify potential illnesses specific to you and your family. For example, information from a blood test could reveal that you have a serious viral disease or a gene that often causes colon cancer. Having a gene (DNA) that indicates future illness increases the possibility that your relatives have the same gene (DNA) and the likelihood of future illness. If you want the individual results of the genetic analyses in the study, you and your relatives may experience mental stress from knowing the genetic (DNA) makeup and possible illnesses in your family. Therefore, when you first confirm your intent to cooperate with this study, please thoroughly consider the benefits and risks that come from knowledge before indicating whether or not you want genetic (DNA) results returned to you. For those who do not want the results in future years, we will return nothing as long as there are no new requests for the results of the

analyses performed in this study.

SHORT FORM

Benefits to Study Participants

■ Results of this study will be publicly announced in specialized academic journals and other media to benefit society in ways such as the development of medical policies, using a format that retains participant privacy.

■ There are absolutely no financial benefits for individual study participants.

■ In addition to providing all health-checkup attendees with the results of supplementary test items determined in consultation with Takashima City, we will also provide study participants with information on waist circumference, insulin levels, high-sensitivity CRP levels, and BNP levels.

■ If results are judged to be significant to medical care or treatment of an individual participant, we will provide the information with an explanation and counseling. This also applies to genetic testing results. However, it may take years to determine whether or not the results are significant for individual

medical care. We will not release the results of a participant who has died.

Risks and Possible Disadvantages from Participating in this Study

▪ There will be no financial burden or cost to study participants.

▪ We will control personal information to prevent external leaks. However, it is impossible to completely eliminate the likelihood that a malicious third party might breach this strictly guarded information, so there is a risk of information theft and leakage.

▪ If theft or a leak of personal medical information does occur, you may incur social risks (eg, discrimination in applying for life insurance or job hunting). The Shiga University of Medical Science and its individual researchers are not responsible for personal information theft or leaks if the researchers are not clearly at fault, so we ask for your understanding of this matter when participating in our study.

▪ In the course of performing a blood test for this study, we may discover information in the future that could affect your health, such as a serious viral disease or a gene that often causes colon cancer. Having a gene that indicates

future illness increases the possibility that your relatives will have the same gene.

■ Consequently, if you want the results of individual genetic analysis, you may experience mental stress from knowing the genetic makeup and possible illnesses in your family.
